# Interferon-α enhances antitumor activities of oncolytic adenovirus-mediated IL-24 expression in hepatocellular carcinoma

**DOI:** 10.1186/1476-4598-11-31

**Published:** 2012-05-08

**Authors:** Cong-Jun Wang, Chao-Wen Xiao, Tian-Geng You, Ya-Xin Zheng, Wei Gao, Zhu-Qing Zhou, Jun Chen, Xin-Bo Xue, Jia Fan, Hui Zhang

**Affiliations:** 1 Department of General Surgery, Shanghai East Hospital, Tongji University School of Medicine, Shanghai, 200120, China; 2 Department of HBPSurgery, The Central Hospital of Wuhan, Tongji Medical College, Huazhong University of Science and Technology, Wuhan, 430014, Hubei Province, China; 3 Liver Cancer Institute and Zhongshan Hospital, Fudan University, Shanghai, 200032, China; 4 Department of Biliary and Pancreatic Surgery, Tongji hospital of Wuhan, Tongji Medical College, Huazhong University of Science and Technology, Wuhan, 430030, Hubei Province, China

**Keywords:** Gene therapy, Hepatocellular carcinoma, Interferon (IFN), Mda-7/IL-24, Nude mice, Oncolytic adenovirus

## Abstract

**Background:**

Hepatocellular carcinoma (HCC) has a dismal 5-year-survival rate of 10%, so novel strategies are warranted. IL-24 mediates anti-tumor activity reducing STAT3 expression, which suggests that interferon (IFN) alpha may augment tumor cell lysis and reduce angiogenesis. We investigated the antitumor activity of treatment with IFN-α, with the oncolytic adenovirus SG600-IL-24, or the combination of both in HCC *in vitro* and *in vivo*.

**Results:**

RT-PCR, ELISA assay and Western-blot confirmed that the exogenous IL-24 gene was highly expressed in HCC cells infected with SG600-IL-24. Treatment with combined IFN-α and SG600-IL-24 suppressed growth and promoted apoptosis of the HepG2, MHCC97L, and HCCLM3 cell lines compared with the normal cell line L02. The combined therapy increased STAT1 and SOCS1 and apoptosis, but decreased the expression of the metastatic and angiogenic proteins MMP-2, XIAP, OPN, and VEGF, which are regulated by STAT3 in HCC cells *in vitro*. To assess the effects *in vivo*, the HCC cell line HCCLM3 was transplanted subcutaneously into the right flanks of nude mice. Mice in the IFN-α group, the SG600-IL-24 group, or the combined therapy group had significantly suppressed growth of the HCC xenografted tumors compared to the PBS control group of mice. Among the mice treated with the combination of IFN-α and SG600-IL-24, three of those eight mice had long-term survival and no evidence of a tumor. These mice also had decreased expression of the metastatic and angiogenic proteins MMP-2, XIAP, OPN, and VEGF.

**Conclusions:**

The present study demonstrated for the first time the potential antitumor activity of IFN-α combined with the oncolytic adenovirus SG600-IL-24 in HCC both *in vitro* and *in vivo*, and suggests its further development as a potential candidate for HCC cancer gene therapy.

## Introduction

Hepatocellular carcinoma (HCC) is strongly associated with chronic infection with hepatitis B virus (HBV), liver cirrhosis, hepatitis C virus infection, aflatoxin exposure, and excessive alcohol consumption [1]. HCC is one of the most common lethal malignancies in the world, and HCC patients have a 1-year survival rate of less than 50% and a 5-year survival rate of 10% [1], despite surgery and chemotherapy [2]. New modalities or combination therapies that increase anti-tumor immune responses and tumor cell lysis are warranted.

The melanoma differentiation associated gene-7 (MDA-7), now known as Interleukin-24 (IL-24), can inhibit the growth and induce the apoptosis of melanoma, along with ovarian [3], lung [4,5], pancreas [6], prostate [7], and colon cancers [8-12]. IL-24 was hailed as a novel and interesting development in experimental tumor therapy in the early 21st century [13]. We have shown that IL-24 can selectively kill several HCC lines *in vitro*[14]. IL-24 mediates its inhibition of metastases and angiogenesis in HCC by suppressing expression of matrix metalloproteinase 2 (MMP-2), vascular endothelial growth factor (VEGF), STAT-3, and phosphorylated STAT3 [14]. To augment efficacy of IL-24, we searched for a complementary anti-tumor protein to deliver in combination therapy. Previous studies suggest that IL-24 selectively kills melanoma cells via an IL-20 receptor-dependent pathway that is independent of STAT3 [15]. In melanoma tumor cells, IL-24 induces IFN-α, which leads to growth inhibition and apoptosis, possibly involving FAS-FASL and TRAIL interactions [16]. Since IFNs promote the activity of STAT1 and SOCS1, but inhibits the activity of STAT3, it is theoretically feasible that IFN-α and IL-24 may augment their anti-tumor activities.

Interferon (IFN) inhibits the activity of tumor cells in many organs and tissues and regulates activities of cytokines which control cell function and replication. IFN may mediate antitumor effects either indirectly by modulating immunomodulatory and anti-angiogenic responses, or directly by affecting proliferation or cellular differentiation of tumor cells. Both the direct and indirect effects of IFNs originate from the activation of the JAK-STAT pathway and the induction of a subset of genes, the IFN-stimulated genes (ISGs) [17]. Administration of high-dose IFN is associated with significant toxicity that includes constitutional, neuropsychiatric, hematologic, and hepatic effects [18]. When INF-α expressed from an oncolytic adenovirus with the *dl*1101/1107 mutation and with overexpressed E3 11.6 K adenovirus death protein was administered to nude mice, efficacy against HCC tumors was improved and toxicity was reduced [19]. This study suggests that co-administering IFN-α with a replication-conditional oncolytic adenovirus may both reduce the toxicity of IFN-α and increase its efficacy, by locally high concentrations of IFN-α at the site of the tumor, while systemic levels remained low. Recent research suggests that interferon significantly improves the effectiveness of chemotherapy, such as 5-fluorouracil and S1 [20,21]. Also, IFN-α delays or reduces the recurrence of liver cancer and improves the overall survival of patients with HBV-related HCC [22]. Furthermore, low miR-26 expression in HCC was associated with greater sensitivity to IFN therapy [23].

Oncolytic adenoviruses, which are gene therapy vectors with intrinsic anti-tumor efficacy, have several beneficial characteristics for their use against liver cancer. First, oncolytic adenoviruses are engineered to selectively replicate and lyze tumor cells but not normal cells [24]. Because the lysed tumor cells release replication-conditional oncolytic adenoviruses that infect surrounding tumor cells, they spread further into the tumor nodules than such standard gene therapy vectors as replication-defective adenoviruses [24]. Tumor-selective replication of oncolytic adenoviruses is dependent on the presence of tumor proteins that complement for the engineered defect in the replication cycle of the oncolytic adenovirus. Fortunately, it is independent of p53 status. Second, adenovirus type 5 specifically binds to hepatocytes via coagulation factor X and heparin sulfate proteoglycans [25]. Replication-competent oncolytic adenovirus SG600-IL-24 has the telomerase reverse transcriptase promoter (TERTp) and the hypoxia regulatory elements (HRE) controlling the expression of adenoviral mutated E1a gene and the E1b, respectively [26-28]. Mutation of the E1a gene and its expression driven by the telomerase promoter hinders its replication in normal cells but not in tumor cells. Therefore SG600-IL-24 has enhanced effectiveness and safety for cancer gene therapy. We have recently shown that SG600-IL-24 inhibits growth and increases apoptosis of several HCC cell lines *in vitro*[27,28]. The *in vitro* anti-tumor activity of oncolytic adenovirus SG600-IL-24 was significantly greater than that of replication defective adenovirus IL-24 [27]. We hypothesized that a three-prong strategy of INF-α, an oncolytic adenovirus, and local expression of IL-24 would be more efficacious than treatment with either IFN-α or an oncolytic adenovirus that expressed IL-24 locally. Here we compared the efficacy of the IFN-α treatment, or intratumoral infection oncolytic adenovirus that expressed IL-24, SG600-IL-24 or both to a PBS control in a HCC xenograft in a nude mouse model. To elucidate potential mechanisms, we also examined the effect of these three treatments and control on the expression of the signaling molecules: STAT1, SOCS1, and STAT3; and on the expression of proteins involved in metastasis and angiogenesis: matrix metalloproteinases 2 (MMP-2), x-linked inhibitor-of-apoptosis protein (XIAP), osteopontin (OPN), and vascular endothelial growth factor (VEGF).

## Results

### SG600-IL-24 mediated ectopic IL-24 expression

**Figure 1:**
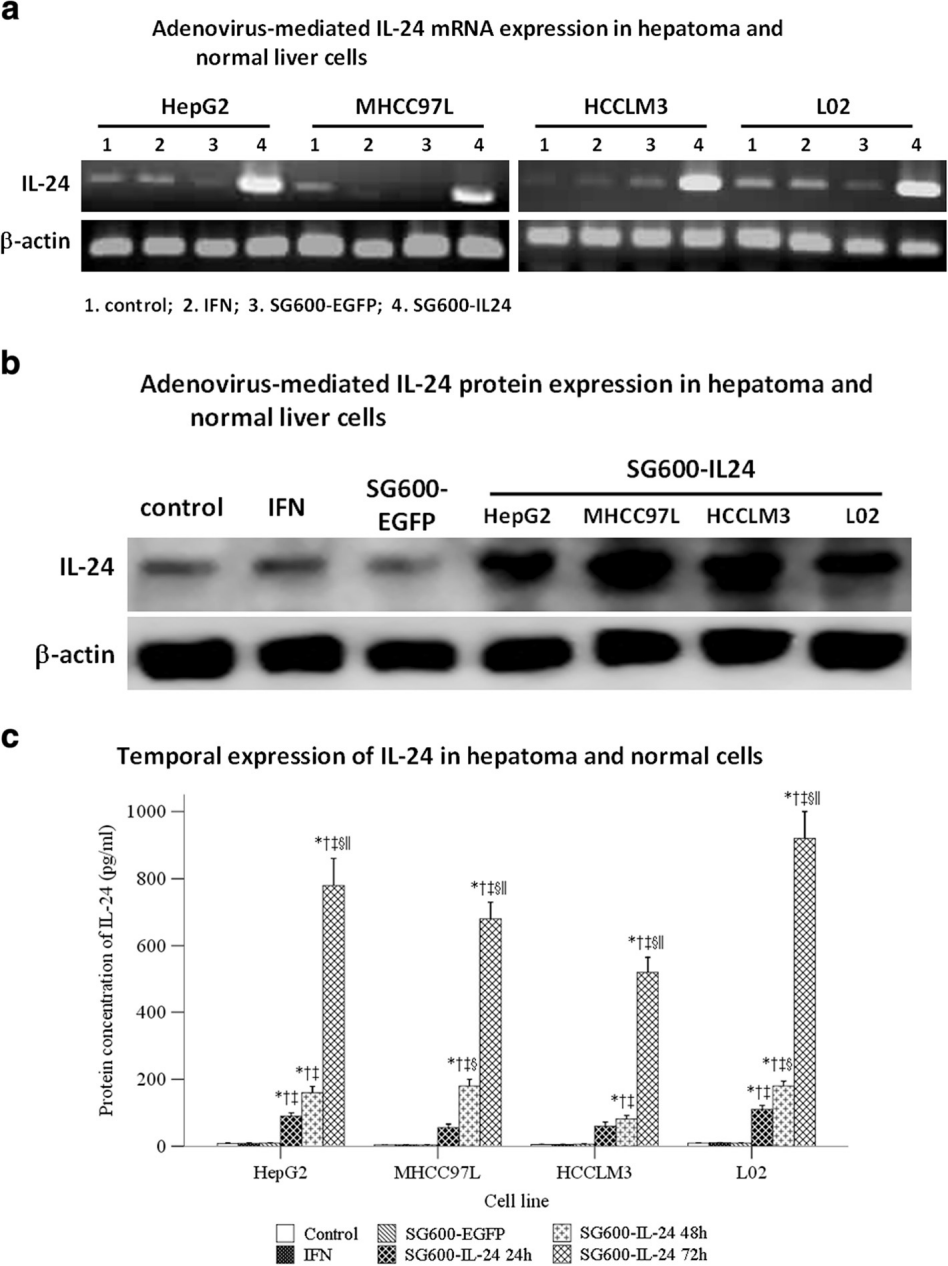
**Expression of adenovirus-mediated interleukin-24 mRNA.** The hepatocellular carcinoma cell lines (HepG2, MHCC97L, HCCLM3) and the human normal liver cell line L02 were treated with control media (Lane 1), treated with 1000 U/ml IFN-α (Lane 2), infected with SG600-EGFP (Lane 3), or infected with SG600-IL-24 at MOI = 10 (Lane 4). **a**. Cells were harvested after 24 h, and RNA was isolated and amplified by RT-PCR as described in “Materials and Methods”. Top line: RT-PCR with IL-24 primers. Bottom line: RT-PCR with β-Actin primers. **b**. Cells were collected 48 h after infection, and protein lysates were isolated and analyzed by western blotting as described in “Materials and Methods”. The HepG2 cell line was used to determine the expression of IL-24 protein after treatment with control, IFN-α, and SG600-EGFP. Top line: IL-24 Bottom line: β-Actin. **c**. Using samples from the supernatant of treated cells, protein expression levels were determined 24 hours after IFN-α treatment, 24 hours after SG600-EGFP treatment, and 24, 48, and 72 hours (as indicated) after SG600-IL-24 treatment.

SG600-IL-24 infection induced higher levels of IL-24 mRNA and protein expression in the normal liver cell line L02, as well as in the HCC cell lines of differing metastatic potential, HepG2, MHCC97L, and HCCLM3. The HepG2 cell line was used to determine the expression of IL-24 protein after treatment with the PBS control, IFN-α, and SG600-EGFP. In contrast, cells treated with IFN-α, DMEM, or infected with the control virus, SG600-EGFP, expressed very low or undetectable concentrations of IL-24 mRNA or protein (Figure 1a, b). Concentrations of IL-24 protein in supernatants of cells treated with SG600-IL-24 significantly increased in a time-dependent manner (Figure 1c).

### Effect of SG600-IL-24 on cell viability

**Figure 2:**
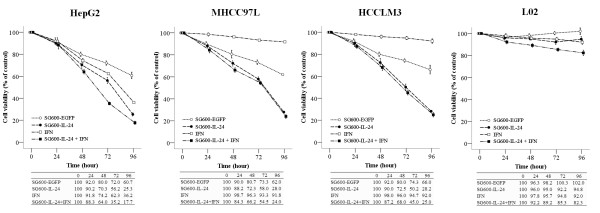
**Cell viability of treated HCC cells and normal liver cells.** HCC cells and normal liver cells were treated as indicated for 24, 48, 72, and 96 hours postinfection, and cell viability was assessed by the MTT assay. Results are presented as mean ± *SD* (n = 5) of five independent experiments and expressed as a percentage relative to the DMEM-treated control cells.

To assess whether the combined IFN-α and SG600-IL-24 treatment was more effective in suppressing cell viability, the hepatocellular carcinoma cell lines HepG2, MHCC97L, and HCCLM3, along with the normal liver cell line L02, were treated as described, and a fifth group was treated with both IFN-α and SG600-IL-24. Cell viability was determined by MTT and compared to the media control. These treatments did not affect the viability of the normal liver cell line L02 (Figure 2); In contrast, the viability of HCC cell lines in the combined IFN-α and SG600-IL-24 treatment group as well as the SG600-IL-24 group were significantly affected.

### IFN-α and SG600-IL-24 induced apoptosis in HCC cell lines but not in a normal liver cell line

**Figure 3:**
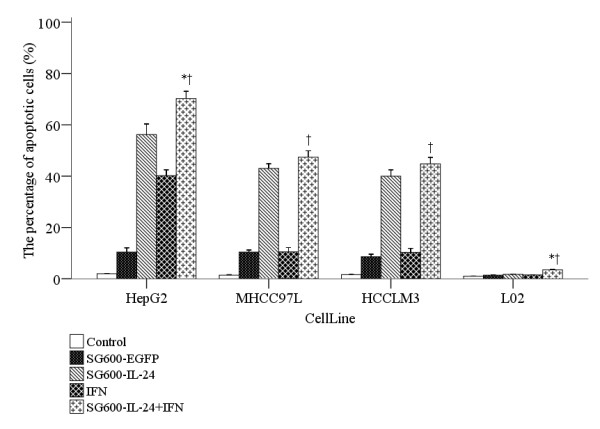
**Combined therapy (IFN-α and SG600-IL-24) boosted apoptosis in HCC lines but not in a normal liver cell line.** Annexin-V and PI staining assays measured the effect of the indicated treatments on 3 HCC lines and the normal liver cell line (LO2). Each group has five independent samples. Results are presented as mean ± *SD*. The symbol * indicates a significant difference compared to the SG600-IL-24 group, and †indicates a significant difference compared to the IFN-α group.

Annexin-V and PI staining assays coupled with flow cytometry quantified the effect of the various treatments on inducting apoptosis in HCC cell lines (HepG2, MHCC97L, and HCCLM3) and the normal liver cell L02 (Figure 3). The proportion of apoptotic HCC cells increased significantly in cells treated with the combination of IFN-α and SG600-IL-24 infected compared with cells treated with control, SG600-EGFP, and IFN-α cells (*p* < 0.018). Both the HepG2 and L02 cells treated with combined IFN-α and SG600-IL-24 had a significantly higher percent of apoptosis than either treatment alone (*p* < 0.01), although it was less than the sum of the effects of both treatments.

### IFN-α + SG600-IL-24 inhibited HCCLM3 cell migration

**Figure 4:**
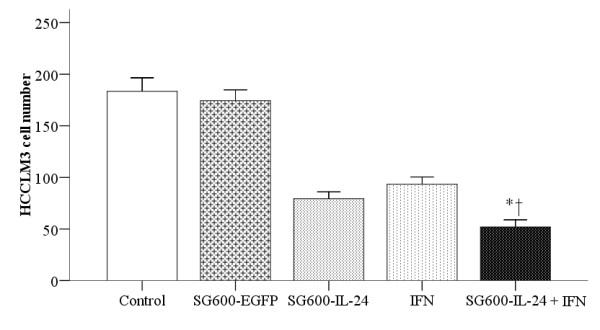
**Combined therapy (IFN**-α **and SG600-IL-24) inhibited HCCLM3 cell invasiveness.** The number of HCCLM3 cells that invaded the extracellular matrix and migrated through the polycarbonate membrane to the lower surface of the membrane was counted to determine migration. The cells were treated as indicated by the column labels. The symbol * indicates a significant difference was observed as compared to the SG600-IL-24 treatment group, and † indicates a significant difference was observed as compared to the IFN-α group.

The transwell assay assessed whether IFN-α or the combination of IFN-α and SG600-IL-24 modulated the process of HCCLM3 cell invasiveness. The HCCLM3 cell line has a high potential for invasion and metastasis compared with other hepatocellular carcinoma cell lines. Migration through matrigel was significantly decreased when HCCLM3 cells were treated with IFN-α, SG600-IL-24, or the combination of IFN-α and SG600-IL-24, compared with that the control or SG600-EGFP groups (*P* < 0.01, Figure 4). Treatment with the combination of both SG600-IL-24 and IFN-α significantly decreased migration compared with either treatment alone (*P*-value = 0.004 and *P*-value < 0.001).

### IFN-α increased STAT1 and SOCS1 but decreased STAT3 in vitro

**Figure 5:**
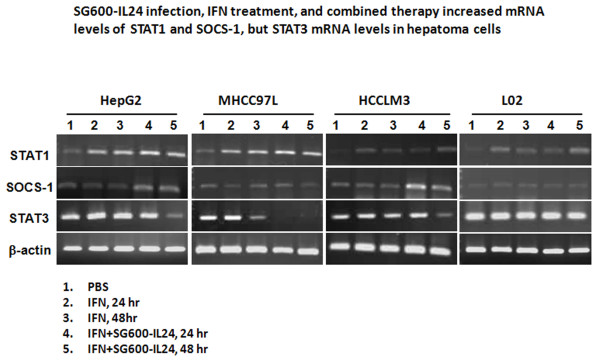
**IFN-αincreased STAT1 and SOCS1 mRNA levels, but decreased STAT3 mRNA in HepG2, MHCC97L, and HCCLM3 cells.** The HCC and L02 cells were cultured with PBS, IFN-α, or both IFN-α and SG600-IL-24 for 48 hours. Then, RT-PCR assessed the mRNA levels of STAT1, SOCS1, and STAT3, along with β-actin, the internal control.

IFN-α signaling modulates expression of STAT1, SOCS1, and STAT3. Since treating HCC cells for 24 or 48 hours with IFN-α slightly increased the expression of STAT1 and had no obvious effects on SOCS1 expression, which was similar to the patterns seen in the normal liver cell line L02, we chose to use the IFN-α treatments for 24 and 48 hours as the background to see the effect of combinations with the virus. We also evaluated the expression of STAT1, SOCS1 and STAT3 in HepG2 and L02 cells treated with SG600-EGFP or SG600-IL-24 for 24 and 48 h (Additional file 1: Figure S5). We showed that combined therapy increased levels of SOCS1 mRNA in HCC cell lines HepG2 and HCCLM3 in a time-dependent manner, compared with either PBS or IFN-α alone (Figure 5). Treatment with IFN-α resulted in decreased STAT3 mRNA levels in the MHCC97L cell line, which was further decreased when the IFN-α treatment was combined with the SG600-IL-24 treatment. While IFN-α did not affect SOCS1 or STAT3 mRNA levels in the normal liver cell L02, IFN-α did increase STAT1 mRNA levels (Figure 5). The data were obtained from three independent experiments.

### MMP and VEGF expression

**Figure 6:**
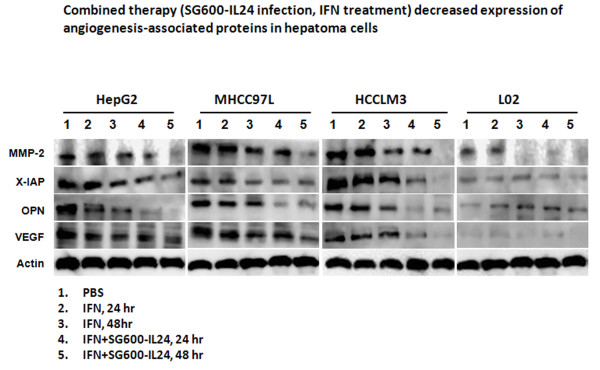
**Expression of MMP-2, XIAP, OPN, and VEGF proteins in IFN-α treated or IFN-αcombined with SG600-IL-24.** Cells from the HepG2, MHCC97L, HCCLM3, and L02 lines were treated for 24 or 48 hours with PBS, IFN-α, or the combination of IFN-α and SG600-IL-24. Then, the relative concentrations of the MMP-2, XIAP, OPN, and VEGF proteins were compared to β-actin on western blots

We analyzed the effect of IFN-α and the combined therapy on expression of MMP-2, XIAP, OPN, and VEGF proteins which are involved in cell invasiveness, metastasis, and angiogenesis (Figure 6). We also included HepG2 and L02 cells infected with SG600-EGFRP and SG600-IL-24 for 24 or 48 h as a control (Additional file 2: Figure S6). IFN-α and the combined therapy decreased protein levels of MMP-2, XIAP, OPN, and VEGF in the HCC lines HepG2, MHCC97L, and HCCLM3 compared with the expression levels of the control group. Both the HepG2 and MHCC97L cell lines had greater decreases in MMP-2, XIAP, and OPN when treated with the combination of IFN-α and SG600-IL-24 than when treated with IFN-α alone. The HCCLM3 cell line had the greatest reductions in the four proteins when it was treated with the combined therapy. L02 cells treated with IFN-α or with the combination of IFN-α and SG600-IL-24 had minimal or no changes in protein levels. The data were obtained from three independent experiments.

### *SG600-IL-24 and IFN*-α *inhibited HCC tumor xenograft growth and prolonged survival time of tumor-bearing mice*

**Figure 7:**
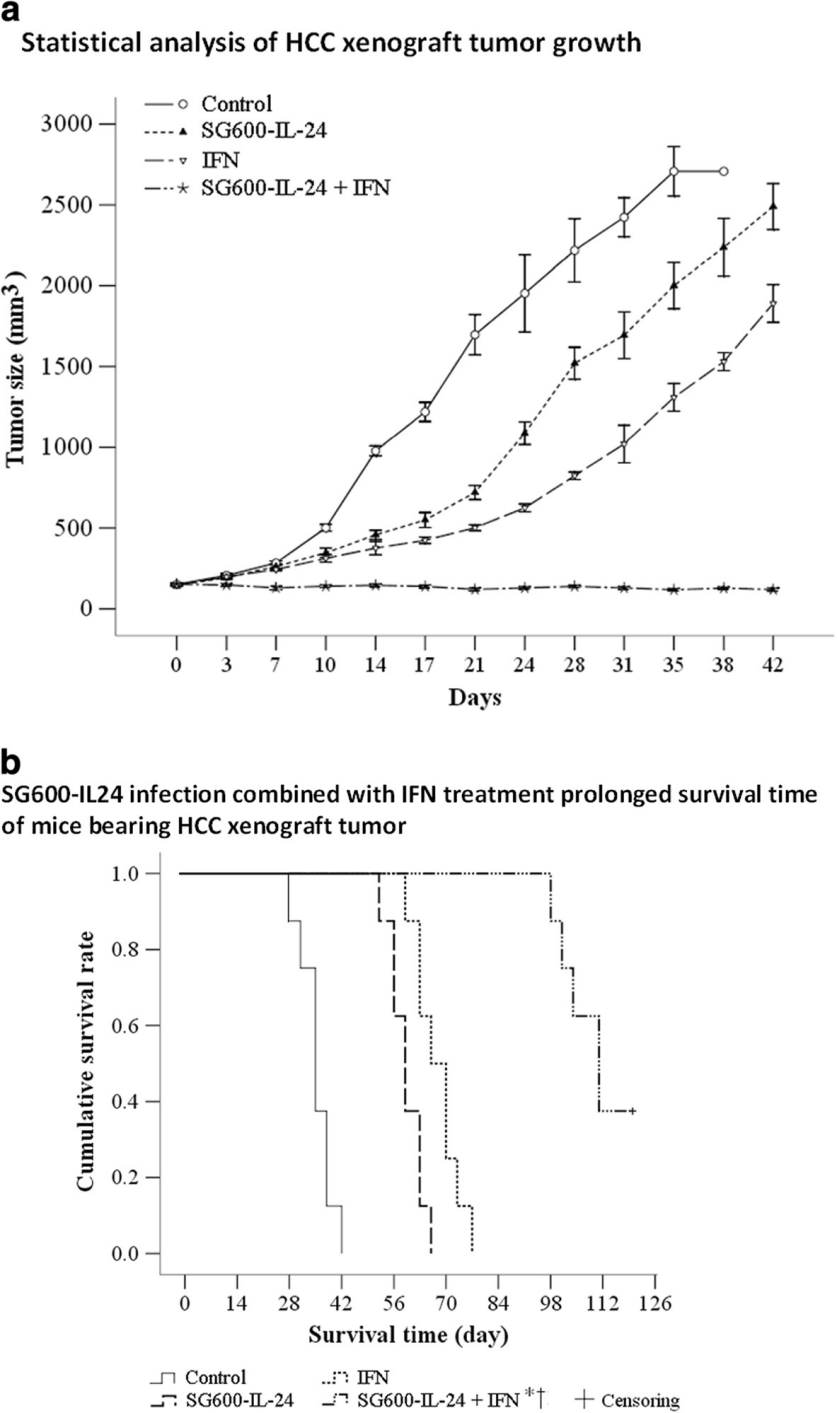
**Antitumor activity of oncolytic adenovirus SG600-IL-24, IFN**-α **and their combination in HCCLM3 xenograft model.** Tumor-bearing mice were treated with PBS, IFN-α daily for 10 days, 2 x 10^8^ PFU of SG600-IL-24 every 2 days for 10 days, or the combination of IFN-α and SG600-IL-24 therapy for 10 days. A, Tumor was measured and volume was calculated, with the time shown from the start of treatment. Data shows mean ± SD (n = 8), though the error bar for the combined treatment group is too small to be clearly identified. B, Kaplan-Meier survival curve. The symbol * indicates a significant difference was observed as compared to the SG600-IL-24 treatment group, and † indicates a significant difference was observed as compared to the IFN-α group

To evaluate the effect of these treatments on tumor growth, we established HCCLM3 xenograft tumors in a nude BALB/c mouse model. Four groups (n = 8 for each group) of tumor-bearing mice were treated with PBS, IFN-α, SG600-IL-24, or the combination of SG600-IL-24 and IFN-α. As expected, the group treated with PBS had progressive tumor growth. Treatment with IFN-α or SG600-IL-24 alone reduced or delayed tumor growth during treatment. However, the combined therapy with SG600-IL-24 and IFN-α resulted in substantial and long-lasting suppression of tumor growth, which was significantly greater than the other treatments (*P* < 0.01; Figure 7a).

Survival times of mice in the IFN-α, SG600-IL-24, and combination groups were significantly prolonged compared with that of the PBS control group (*P* < 0.01; Figure 7b). The median survival times were 35 days for the control group, 59 days for the SG600-IL-24 group, and 66 days for the IFN-α group. In comparison, the median survival time of the combination group (SG600-IL-24 + IFN-α) was 111 days and three of the eight animals survived over 120 days with no evidence of tumor (Figure 7b). Importantly, RT-PCR and immunoblotting demonstrated high levels of IL-24 mRNA and protein in tumor tissue of treated mice even at 4 weeks after infection with the oncolytic viruses (Additional file 3: for viral existence).

**Figure 8:**
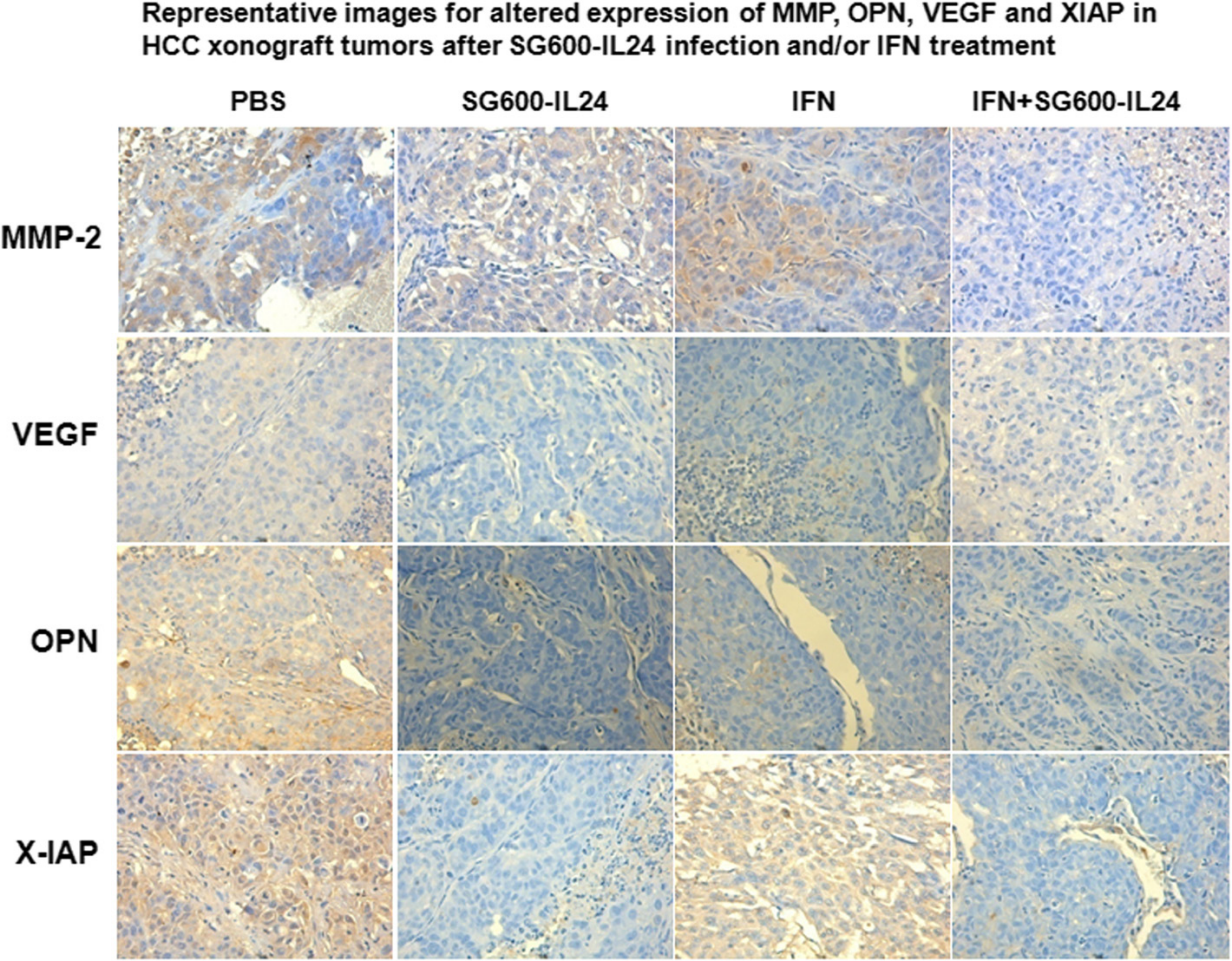
**Expression of MMP-2, OPN, VEGF, and XIAP in tumor tissue.** Tumor tissues from five different mice from each group were stained for MMP-2, OPN, VEGF, and XIAP expression by immunohistochemistry and visualized with a E80i microscope (Nikon Corporation, Japan) under 200x magnification

To begin to elucidate mechanisms mediating the improved efficacy of the combined therapy group, we assessed the expression of several proteins involved in invasiveness and angiogenesis (MMP-2, XIAP, OPN, and VEGF) in the tumor tissue of the treated groups. As aforementioned, tumor tissue was only present in five of the eight mice treated with the combined therapy. The percent of tumor cells positive for MMP-2 expression was significantly decreased in the INF-α group compared with the controls, and the MMP-2 levels in the combined therapy group were further significantly reduced compared to the IFN-α, control, and IL-24 groups (Figure 8, Table 1). The percent of cells positive for OPN and XIAP expression was significantly reduced in the IL-24 group and the combined therapy group (Figure 8, Table 1). The percent positive cells for VEGF expression was significantly reduced by the three treatments (IL-24, IFN-α, and the combined therapy) (Figure 8, Table 1). These data indicated that the combined therapy mediated its anti-tumor activity via multiple pathways. Notably, the human IFN-α does not cross-react with the mouse IFN receptor, and the human IL-24 expressed by the virus does not cross-react with the mouse IL-24 receptor. Both human and mouse IFN-α can be detected by the ELISA kit.

**Table 1:** Statistical analysis of immunohistochemistry on treated tumor tissues

	**Control(n = 5 mice)**	**IL-24(n = 5 mice)**	**IFN-α(n = 5 mice)**	**IL-24 and IFN-α(n = 5 mice)**	**P-value**
MMP	74.0 ± 8.5	66.2 ± 10.2	48.0 ± 10.3*	2.0 ± 1.9*†‡	<0.001
OPN	68.0 ± 12.8	0.6 ± 0.9*	9.2 ± 3.7*†	0.6 ± 0.5*‡	<0.001
VEGF	17.8 ± 4.7	1.6 ± 1.1*	1.4 ± 1.5*	0.8 ± 1.1*	<0.001
XIAP	90.8 ± 3.6	0.8 ± 0.4*	79.0 ± 7.0†	0.6 ± 0.5*‡	<0.001

* significant difference compared to the control group,

† significant difference compared to the IL-24 group.

‡ significant difference compared to the IFN-α group.

### IFN-α expression

To further elucidate the role of IFN-α, we assessed the serum concentrations of IFN-α in the treated groups compared to that of the control group. Blood samples were collected from the four groups of mice after treatment for a week followed by serum separation and stored in −20°C. Serum concentrations of IFN-α were 57 ± 23 pg/ml in the control group, 280 ± 35 pg/ml in the IFN-α group, 46 ± 22 pg/ml in the SG600-IL-24 group, and 292 ± 52 pg/ml in the combined therapy group.

## Discussion

Liver cancer gene therapy is a major research focus because the 5-year survival for liver cancer patients is 10%, despite considerable advances in multimodality treatment that include surgery, radiotherapy, and chemotherapy [1]. Oncolytic adenoviruses have an intrinsic specificity for liver cancer cells [25]. Recent advances in vector development have improved tumor specificity of its replication [26], in agreement with our data in HCC lines. Infection with SG600-IL-24 displayed CMV-driven IL-24 production in both normal cells L02 and in the 3 HCC cell lines. In contrast, viral induced lysis was detectable only in the 3 HCC cell lines. Our observed secretion of IL-24 can also contribute to the potent “bystander” antitumor activity [29] and may target micrometastases [14].

Comparable IL-24-induced anti-tumor activity *in vitro* was observed in the three HCC cell lines which differed by their metastatic potential; these data further supported the findings that tumors which are sensitive to mitochondrial dysfunction and apoptosis induced by SG600-IL-24 have a range of defects in various proteins including p53, p16/INK4a, and Rb [4,30,31]. The cell line HepG2 expresses wild type p53 but has a mutant Rb, while the cell lines MHCC97L and HCCLM3 have mutant p53 [32-35]. The significantly improved median survival time, the 37% long term survivors, and the significantly lower tumor volume in the combined therapy (SG600-IL-24 and IFN-α) compared to those of the single modality groups and controls suggests that these modalities complement their anti-tumor activities. We have just begun to elucidate mechanisms for this interaction. It is important to note that the lack of IFN-α toxicity in mice as a result of systemic administration in this study could be due to species specificity of the IFN-α receptor. This needs to be kept in mind while extrapolating these data to humans.

The SG600-IL-24 virus selectively lyses melanoma cells via an IL-20 receptor-dependent mechanism which is independent of the STAT3 pathway [15]. It is feasible that the SG600-IL-24 virus mediates its anti-tumor activity via STAT3 inhibition. While IFN-α can promote the activity of STAT1 and SOCS1 definitely, IFN-α inhibits the activity of STAT3, in accordance with these studies. Activating STAT3 is necessary for the VEGF receptor signaling pathway in endothelial cells [36,37]. Blocking the expression of STAT3 inhibits the migration and lumen formation of endothelial cells [37]. Persistent STAT3 activity promotes *in vivo* angiogenesis, in part by inducing VEGF, a potent inducer of angiogenesis [38,39]. Persistent activated STAT3 also stimulates invasiveness and metastasis by inducing MMP-2 *in vitro* and *in vivo*[39]. Thus, inhibition of aberrant STAT3 suppresses VEGF expression and angiogenesis [38,39]. XIAP is the most potent caspase inhibitor [40], and it is increased in HCC [41]. High XIAP are associated with reduced survival [42]. High intracellular OPN regulates cell survival and is essential both for IFN-α production by plasmacytoid dendritic cells [43] and for the migration of metastatic tumor cells [44]. Our results suggest that IFN-α or combined therapy with SG600-IL-24 inhibited the levels of STAT3 in HCC cell lines, and reduced levels of downstream proteins including MMP-2, XIAP, OPN, and VEGF. This led to increased rates of apoptosis in hepatoma cells and significantly decreased metastatic potential.

Although increasing chemotherapeutic drug concentrations causes greater tumor cell death, harmful effects are also seen in normal cells, which may lead to failure of therapy failure because the treatment can be intolerable for patients. Therefore, approaches are critically needed that maximize the chemotherapeutic effect and allow both the drug dosage and the length of treatment to be reduced. Previous studies suggest that IL-24 and radiotherapy synergistically destroy tumors, inhibit tumor growth, and reduce intratumor angiogenesis [45,46]. We hypothesized that IFN-α combined with the oncolytic adenovirus SG600-IL-24 would provide greater efficacy against HCC. Our results demonstrated that the combined therapy had a significantly smaller mean tumor volume and increased survival time, with three of eight mice being long-term survivors. Thus, the dosage can be decreased and the corresponding toxicity reduced. The viruses continue to exist in the tumors, as the oncolytic adenovirus exists at both the mRNA and protein levels four weeks after infection [47].

Notably, we determined the specific activity of the serum IFN-α by ELISA, where standards are prepared in pg/ml. Commercialized IFN-α preparations are sold with activity described based on a viral resistance assay that uses bovine kidney MDBK cells to define IU/mg. Our study did not test the bioactivity of the serum IFN-α, so our study is limited by our inability to correlate the levels detected (expressed in pg/ml) to the doses administered (expressed in IU/mg). Additionally, the effects observed when we combined IFN-α with SG600-IL-24 *in vitro* differed from those observed *in vivo*. The anti-tumor activity of IL-24 was previously shown to be mediated by an IL-20 receptor-dependent but STAT3-independent mechanism [15]. Furthermore, INF-α was shown to activate of STAT1 and SOCS1 while inhibiting STAT3 in order to promote angiogenesis [38,39]. Our mRNA and protein expression measurements (Figure 5 and 6) were therefore designed based on IFN-α signaling and did not include an IL-24 group. Our goal was to understand if combining IFN-α with IL-24 could enhance anti-tumor activity via inhibition of STAT3 to promote anti-angiogenic effects.

In summary, this study demonstrated that combining SG600-IL-24 and IFN-α resulted in significantly longer mean survival time, along with lower levels of the proteins VEGF, OPN, STAT3, and XIAP. These findings suggest that targeting gene-virotherapy combined with IFN-α can provide an efficient strategy in HCC cancer treatment and warrants further development.

## Materials and methods

### Cell lines, culture conditions, reagents and mice

The HCC cell lines (HepG2, MHCC97L, and HCCLM3) and the normal human liver cell line L02 were purchased from the Institute of HCC of Fudan University. Cell lines were cultured in high glucose DMEM (HyClone, USA) supplemented with 10% FBS (Gibco, USA). L02 cells were cultured in RPMI-1640 (HyClone, USA) supplemented with 10% FBS. All cell lines were cultured at 37°C in a 5% CO_2_ humidified incubator.

### Virus construction and production

The oncolytic adenovirus which expressed enhanced green fluorescent protein, SG600-EGFP was constructed and amplified as previously described for SG600-IL-24 [28]. The SG600 adenovirus is regulated by the hTERT promoter and is replication competent. The adenoviral E1a gene is driven by the hTERT promoter sequence. The SG600 adenovirus can selectively replicate in a broad array of human cancer cells with positive telomerase activity, but not in normal cells. Both IL-24 and EGFP were driven by the CMV promoter. Genomes were analyzed to confirm the recombinant structure. Due to nomenclature change, SG600.mda-7 was renamed SG600-IL-24. The virus was plaque purified, amplified in HEK293 cells, and stored in aliquots at −80°C. Viral titers were determined by TCID50 assay in HEK293 cells. The titers were 2.25 × 10^10^ plaque-forming units (PFU)/ml for SG600-IL-24, and 2.79 × 10^10^ PFU/ml for SG600-EGFP.

### Viral infection and IFN-α treatment

Six-well plates for each cell line were divided into 4 groups: control, IFN-α, SG600-EGFP, and SG600-IL-24 groups. The control group was incubated with serum-free DMEM for 24 h. Cells in the SG600-EGFP and SG600-IL-24 groups were infected with their respective viruses at a multiplicity of infection (MOI) of 10. The IFN-α group was incubated with 1000 U/ml of IFN-α (Sigma, St. Louis, MO, USA) for the same time period.

### RT-PCR

To quantitate IL-24 RNA, cells were infected with SG600-IL-24 (MOI = 10), incubated 24 h, and harvested. Total RNA was extracted and RT-PCR was performed as previously described [28,48]. Primer sequences of IL-24 mRNA (sense: 5'-GGG CTG TGA AAG ACA CTA T-3'; antisense: 5'-GCA TCC AGG TCA GAA GAA-3') amplified a 381 bp fragment. Primer sequences of β-Actin (sense: 5'-CCT TCC TGG GCA ATG GAG TCC T-3'; antisense, 5'-GGA ACA ATG ATC TTG ATC TT-3') amplified a 201 bp fragment. PCR conditions (RT-PCR kit (TAKARA, JPN)) were denaturation at 94°C for 5 min; 30 cycles of denaturation at 94°C for 30 s, annealing at 56°C for 30 s, and extension at 72°C for 45 s; and extension at 72°C for 10 min to ensure full extension of the product. The amplified products were visualized by electrophoresis on a 1% agarose gel. To quantitate apoptotic genes, cells were treated with PBS, IFN-α (1000 U/ml), or IFN-α combined with SG600-IL-24 (MOI = 10). We harvested the cells and RNA in the PBS group after 24 h, and the cells treated with IFN-α group or the combined therapy at 24 and 48 h. Primer sequences for STAT1 (sense: 5'- TTCTGGCCTTGGATTGAC-3'; antisense, 5'-TCTCAGCAGCCATGACTT-3') amplified a 313 bp fragment; SOCS1 primers (sense, 5'- 5’-AGACCCCTTCTCACCTCTTG-3'; antisense, 5'-CTGCACAGCAGAAAATAAAGC -3') amplified a 202 bp fragment; and STAT3 primers (sense, 5'- CGTCCAGTTCACTACTAAAGTCAGG -3', antisense, 5'- CTCAGTCACAATCAGGGAAGCA -3') amplified a 277 bp fragment.

### ELISA

Cell culture supernatants were collected at 24, 48, and 72 h and stored at −20°C. Concentrations of IL-24 were determined by IL-24 ELISA (R&D Systems, Minneapolis, MN, USA) using a standard curve. Absorbance was read at 450 nm. Serum concentrations of IFN-α in the tumor-bearing mice were measured using an ELISA kit (R&D Systems, Minneapolis, MN, USA).

### MTT assay to determine cell growth

A total of 10^3^ cells were seeded into 96-well tissue culture plates, and treated with PBS, 1000 U/ml IFN-α,10 MOI of SG600-EGFP, 10 MOI of SG600-IL-24, or both 1000 U/ml IFN-α and 10 MOI of SG600-IL-24. After the indicated times, media was removed, and fresh medium with 0.5 mg/ml MTT (Sigma, St. Louis, MO, USA) was added to each well. After cells were incubated at 37°C for 4 h, supernatant was removed, 150 μl dimethylsulfoxide was added to each well, and the cells were incubated for another 10 min at 37°C with gentle shaking. Absorbance was read on a Bio-Rad microplate reader at 595 nm [24].

### Western blot analysis

Cell lines were cultured in six-well plates, treated with the different treatments, and collected at the indicated times. Next, the cells were suspended in RIPA lysis buffer that contained protease inhibitors and then quantitated by the BCA method. To quantitate IL-24, 25 μg protein samples were separated by 15% SDS-PAGE and then transferred to polyvinylidene fluoride (PVDF) membranes. The PVDF membranes were probed with polyclonal antibodies either to mda-7/IL-24 (Abcam, UK) or to β-actin, then corresponding fluorescent secondary antibody was added, and then the fluorescence signal was detected with the infrared imaging systems. To quantitate apoptotic genes, 50 μg protein samples were separated on an 8-15% SDS-PAGE gel and transferred to PVDF membranes. The PVDF membranes were probed with polyclonal or monoclonal antibodies to STAT1, SOCS1, STAT3, MMP-2, XIAP, OPN, VEGF, and β-actin (Thermo Scientific, Lab Vision, CA, USA). The blots were evaluated with enhanced chemiluminescence (ECL), and β-actin was detected on the same membrane and used as a loading control.

### Hoechst33258 staining

Cells were infected, incubated for 48 h, washed twice with PBS, and fixed in 1 ml of 4% paraformaldehyde for 10 min at 4°C. After two washes with PBS, cells were stained with 100 μl Hoechst33258 (Sigma) in PBS for 15 min in the dark at room temperature, and then 1000 stained cells were examined for nuclear fragmentation with a TE2000-U fluorescence microscope (Nikon, Japan). Apoptotic cells were identified by the nuclear chromatin condensing and fragmenting.

### Fluorescence-activated cell sorter (FACS) analysis

Cells were harvested 48 h after treatment, trypsinized, and washed twice with complete media. A total of 10^6^ cells were resuspended in 500 μl binding buffer and stained with 5 μl fluorescein isothiocyanate (FITC)-labeled Annexin-V (Annexin-V/PI kit, Sigma). A total of 0.25 μg propidium iodide (PI) was added to the samples after staining with Annexin-V, and then the samples were incubated in the dark for 30 min. Flow cytometry (BD, FACSCalibur, USA) was performed immediately after staining.

### Transwell assay

Cell migration analysis was performed by using a Transwell (Corning, NY). The transwell chamber containing an 8-μm pore size polycarbonate membrane filter coated with a matrigel (16.5μL/membrane filter, BD Bioscience, Bedford, MA) was inserted in a 24-well culture plate. Cells were infected and harvested after 48 h. A total of 100μL of 2 × 10^5^ cells were placed in the upper transwell chamber in serum-free DMEM high glucose culture medium. A total of 600 μL of high glucose DMEM media containing 10% fetal bovine serum was added into the lower transwell chamber. After reculturing with 5% CO_2_ at 37°C for 20 h, the transwell chambers were inverted and stained with Giemsa for 15 min. Five fields were randomly selected and the number of trans-membrane cells in those fields was counted.

### Animal studies

Male BALB/c nude mice at 4 to 6 weeks of age were obtained from Liver Cancer Institute of Zhongshan Hospital, Fudan University (Shanghai, China) and maintained in pathogen-free conditions in accordance with the National Institutes of Health Guide for the Care and Use of Laboratory Animals. A total of 2 × 10^6^ HCCLM3 cells were injected subcutaneously (s.c.) into the right flanks of mice. When tumors reached 100–150 mm^3^, mice were randomly divided into four treatment groups (n = 8 each): control (saline injection), IFN-α alone (1.5 × 10^4^ IU/g of human IFN-α, intraperitoneal (i.p.) injection, once per day for 10 days), SG600-IL-24 alone (2 × 10^8^ PFU, 5 intratumor (i.t.) injections on 2 day intervals for 10 days), or combination (SG600-IL-24 with 2 × 10^8^ PFU and also IFN-α with 1.5 × 10^4^ IU/g, both over 10 days). Tumor length and width was measured twice weekly, and tumor volume was calculated as follows: Tumor volume = length x width^2^/2. Survival time was also monitored with a survival of more than 120 days considered long-term survival.

### Immunohistochemical analysis of tumors

Tumors of the five mice from each treatment group were harvested on day 30 for hematoxylin/eosin staining and examined for tumor cell differentiation. We performed immunohistochemical staining to detect expression of MMP-2, X-IAP, osteopontin, and VEGF. Formalin-fixed tissue sections that were 5 μm wide were mounted on microscope slides, dried overnight at 60°C, dewaxed in xylene, and rehydrated with distilled water. Endogenous peroxidase was quenched by treating the sections with 1% H2O2 in methanol for 10 min. Sections were incubated overnight at 4°C with 1:500 dilutions of rabbit polyclonal antibody to MMP-2, X-IAP, osteopontin, and VEGF (Thermo Scientific, Lab Vision, CA, USA), washed with PBS three times, and then incubated with the appropriate secondary antibody (Santa Cruz Biotech). After washing in PBS three times, the sections were treated with DAB substrate and hematoxylin as a counterstaining reagent. To determine the percentage of positive cells, at least 1000 cells/slide were counted and scored by an E80i microscope (Nikon, Japan) under 200× magnification.

### Statistical analysis

All analyses were performed with SPSS14.0 software. The experiments were performed at least three times. Results are expressed as mean ± standard deviation (*SD*). Statistical comparisons were made using analysis of variance (*ANOVA*). Values of *p* < 0.05 were considered statistically significant.

## Competing interests

The authors declare that they have no competing interests.

## Authors’ contributions

We declare that all the listed authors have participated actively in the study and all meet the requirements of the authorship. Dr. Cong-Jun Wang, Dr. Jia Fan, and Dr. Hui Zhang designed the study and wrote the protocol, Dr. Xin-Bo Xue contributed administrative, technical, or material support, Dr. Wei Gao, Dr. Chao-Wen Xiao managed the literature searches and analyses, Dr. Zhu-Qing Zhou undertook the statistical analysis, Dr. Tian-Geng You wrote the first draft of the manuscript, Dr. Ya-Xin Zheng, and Dr. Jun Chen provide critical versions of the manuscript for important intellectual content. All authors read and approved the final manuscript.

## Supplementary Material

Additional file 1 **Additional file 1**. Supplementary figure for figure 5. Click here for file

Additional file 2 **Additional file 2**. Supplementary figure for figure 6. Click here for file

Additional file 3 **Additional file 3**. Supplementary figure for virus existence. Click here for file
