# Supplementary figures and images for: Interferon-α enhances antitumor activities of oncolytic adenovirus-mediated IL-24 expression in hepatocellular carcinoma
Source: Mol Cancer. 2012 May 8;11:31. doi: 10.1186/1476-4598-11-31 (PMC3697897; doi:10.1186/1476-4598-11-31)

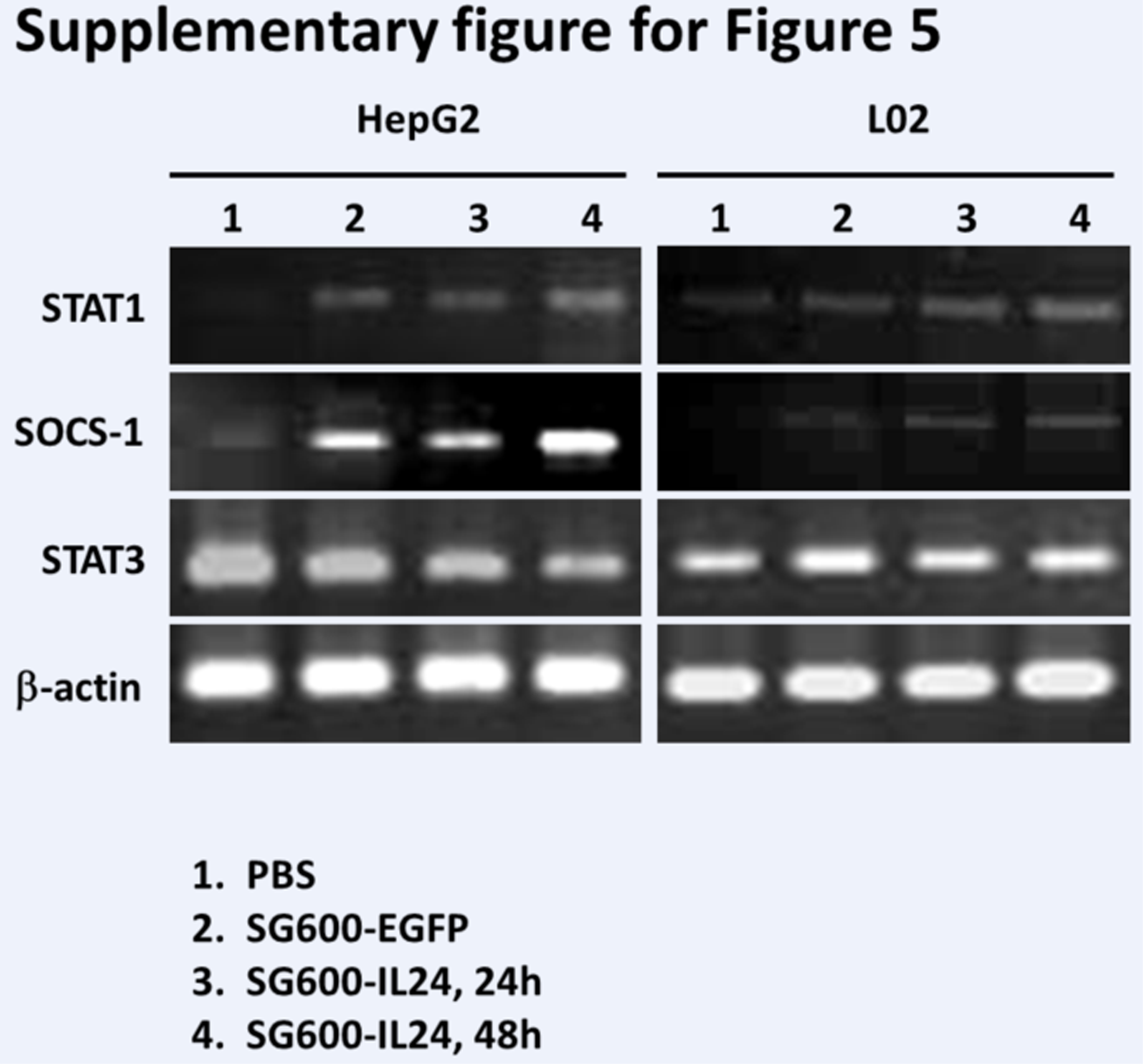

Supplement: Additional file 1 — Supplementary figure for figure 5. [file 1476-4598-11-31-S1.tiff]

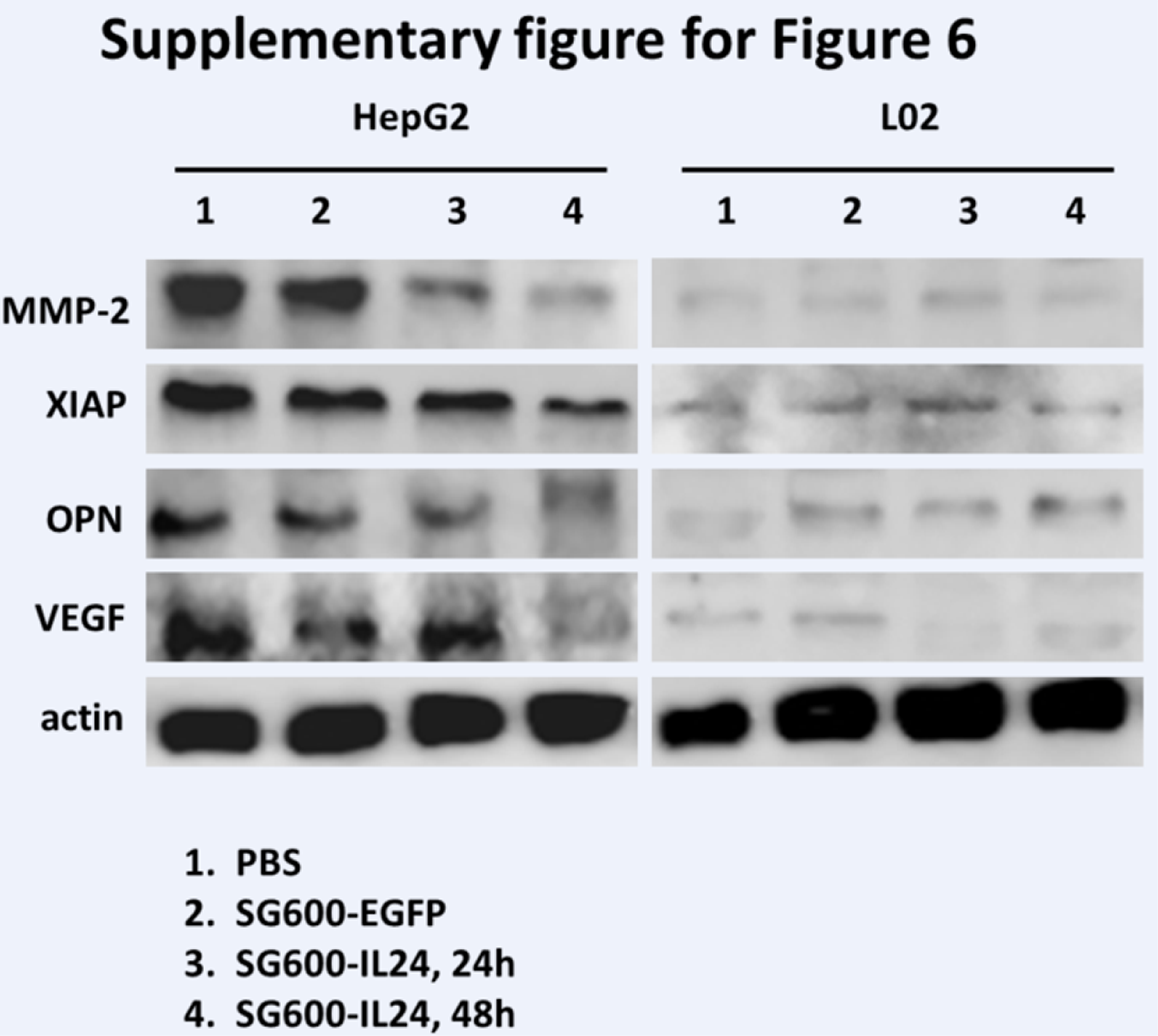

Supplement: Additional file 2 — Supplementary figure for figure 6. [file 1476-4598-11-31-S2.tiff]

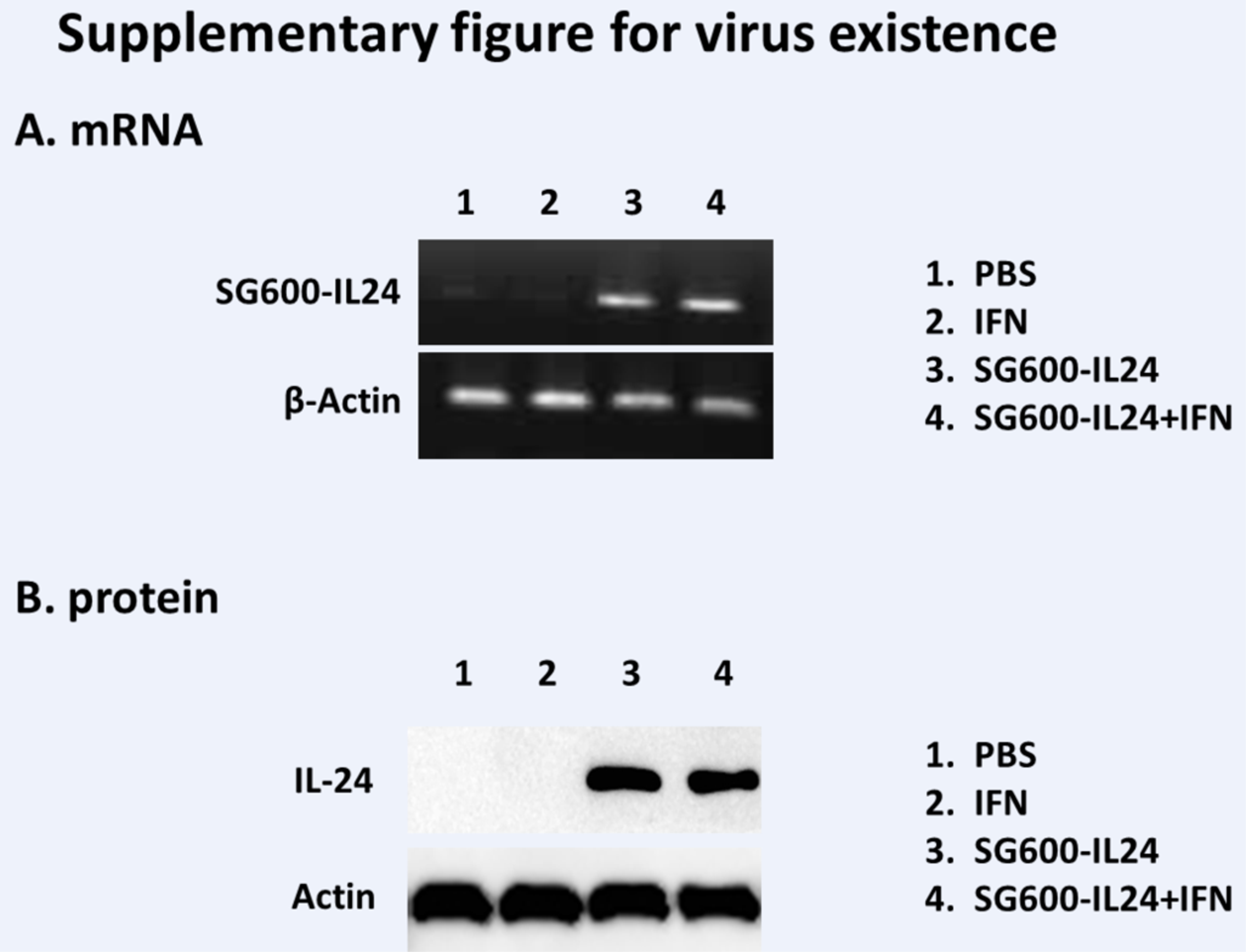

Supplement: Additional file 3 — Supplementary figure for virus existence. [file 1476-4598-11-31-S3.tiff]
